# Patterns of brain asymmetry associated with polygenic risks for autism and schizophrenia implicate language and executive functions but not brain masculinization

**DOI:** 10.1038/s41380-021-01204-z

**Published:** 2021-07-01

**Authors:** Zhiqiang Sha, Dick Schijven, Clyde Francks

**Affiliations:** 1 Language and Genetics Department, Max Planck Institute for Psycholinguistics, Nijmegen, The Netherlands; 2 Donders Institute for Brain, Cognition and Behaviour, Radboud University, Nijmegen, The Netherlands

**Keywords:** Autism spectrum disorders, Schizophrenia, Genetics, Neuroscience

## Abstract

Autism spectrum disorder (ASD) and schizophrenia have been conceived as partly opposing disorders in terms of systemizing vs. empathizing cognitive styles, with resemblances to male vs. female average sex differences. Left–right asymmetry of the brain is an important aspect of its organization that shows average differences between the sexes and can be altered in both ASD and schizophrenia. Here we mapped multivariate associations of polygenic risk scores for ASD and schizophrenia with asymmetries of regional cerebral cortical surface area, thickness, and subcortical volume measures in 32,256 participants from the UK Biobank. Polygenic risks for the two disorders were positively correlated (*r* = 0.08, *p* = 7.13 × 10^−50^) and both were higher in females compared to males, consistent with biased participation against higher-risk males. Each polygenic risk score was associated with multivariate brain asymmetry after adjusting for sex, ASD *r* = 0.03, *p* = 2.17 × 10^−9^, and schizophrenia *r* = 0.04, *p* = 2.61 × 10^−11^, but the multivariate patterns were mostly distinct for the two polygenic risks and neither resembled average sex differences. Annotation based on meta-analyzed functional imaging data showed that both polygenic risks were associated with asymmetries of regions important for language and executive functions, consistent with behavioral associations that arose in phenome-wide association analysis. Overall, the results indicate that distinct patterns of subtly altered brain asymmetry may be functionally relevant manifestations of polygenic risks for ASD and schizophrenia, but do not support brain masculinization or feminization in their etiologies.

## Introduction

Autism spectrum disorder (ASD) is a childhood-onset disorder that features deficits in social communication and social interaction, together with restricted or repetitive behavior [1]. The cognitive and behavioral profile of ASD has been conceived in terms of over-systemizing (focusing heavily on the component variables of systems) and under-empathizing (a deficit in recognizing the thoughts and emotions of others) [2]. A similar concept involves over-mechanizing in ASD (a focus on interaction with the physical environment) and under-mentalizing (deficits in attributing mental states to others) [3]. Given that average sex differences also exist in these dimensions, with males tending to outperform on systemizing tasks and underperform on empathizing tasks, ASD was proposed to resemble excessive masculinization of the brain [2].

In contrast, positive symptoms in schizophrenia, which can involve the incorrect attribution of thoughts or feelings to external agents, have been conceived in terms of hyper-mentalizing [3–6]. This has led to models of psychosis and ASD as opposing disorders of the social brain that partly mirror average sex differences [3–7].

There are also features often found in common between ASD and schizophrenia, including impaired executive functions [8–11] and deficits in social cognition and competence in the verbal and non-verbal domains [12, 13]. However, such similarities may be relatively superficial and not reflect etiological commonalities [12, 14, 15]. Large-scale cross-disorder analyses have pointed to shared genetic contributions to ASD and schizophrenia, but although these have been particularly evident for rare mutations of relatively high penetrance [16, 17], the genetic correlation based on common single nucleotide polymorphisms (SNPs) has been estimated at only roughly 0.2 or less [18]. Therefore, both shared and independent genetic contributions are involved.

Left–right asymmetry is a pervasive organizing principle of the human brain’s structure and function [19, 20], including for networks involved in language and social cognition [21–24], and executive [25] and affective processes [26, 27]. Various aspects of hemispheric asymmetry can be altered in ASD or schizophrenia, which include regional anatomical measures of gray and white matter, structural and functional connectivity, and behavioral associations (non-right-handedness has an elevated frequency in both disorders compared to the general population) [28–41]. Some structural and functional measures of hemispheric asymmetry also show average differences between the sexes [42–44] and fetal testosterone levels relate to the development of gray matter asymmetries of some cerebral cortical regions [45]. Population-average hemispheric asymmetries are established prenatally [46, 47], likely through a genetically regulated program [48–50], and variation in some aspects of brain structural asymmetry is significantly heritable [42, 43]. Therefore, brain asymmetry presents a potential intermediate phenotype between genes and diagnosis, which can help to identify etiologic similarities and differences between ASD and schizophrenia, including with respect to masculinization vs. feminization.

In 32,256 adult participants from the general population UK Biobank dataset, we previously found that 42 regional brain asymmetry indexes showed significant SNP-based heritabilities ranging from 2.2% to 9.4% [51]. These comprised 28 cortical regional surface area asymmetries, 8 cortical thickness asymmetries, and 6 subcortical volume asymmetries, where the asymmetry index for a given region and individual was calculated as (Left − Right)/((Left + Right)/2) [51]. Multivariate genome-wide association scanning of these asymmetries implicated genes involved in microtubule-related functions and genes particularly expressed in the embryonic and fetal brain [51]. These observations are compatible with known roles of the cytoskeleton in shaping cellular chirality, which can initiate left–right asymmetry in the development of other organs of other species [52–57]. SNPs that showed relatively low multivariate association *p*-values in relation to brain asymmetries [51] also tended to show low *p*-values in publicly available genome-wide association summary statistics for ASD and schizophrenia, which indicates a genetic overlap of brain asymmetries with these disorders.

Substantial SNP-based heritabilities for both ASD and schizophrenia indicate that they often arise at the severe tail of a continuous scale of liability across the general population [58]. One way to approach the etiologies of these disorders is therefore to study associations of polygenic risk with brain structure and function in general population datasets. In this approach, the polygenic risk of a given disorder for each individual within a population cohort is estimated from their own genotypes, in combination with SNP-wise summary statistics from large-scale genome-wide association scanning for that disorder [59–64].

Here we calculated polygenic risk scores for ASD and schizophrenia for each of 32,256 individuals from the UK Biobank brain imaging dataset, by making use of the UK Biobank genotype data in combination with summary statistics from previous genome-wide association scans of ASD [29] and schizophrenia [35]. We used canonical correlation analysis to test the associations of ASD and schizophrenia polygenic risks with multivariate brain structural asymmetry, measured using 42 regional asymmetry indexes. The patterns of multivariate association of these two disorder polygenic risks with brain asymmetry were compared against each other, by correlating the loadings across the 42 asymmetry indexes. We functionally annotated the brain regions showing the strongest associations of their asymmetries with each disorder polygenic risk, through querying meta-analyzed data from functional magnetic resonance imaging (MRI) studies. We then investigated whether polygenic risk for either disorder was associated with a more male-like or female-like multivariate pattern of brain asymmetry. Furthermore, we tested for associations of the two disorder polygenic risks with handedness across individuals, followed by phenome-wide association analyses to understand which other brain, behavioral, clinical, and other types of variables are related to polygenic risks for these disorders.

## Methods

### Participants

The UK Biobank is a general adult population cohort [65]. Data availability and processing (below) resulted in 32,256 participants (15,288 male and 16,968 female) with polygenic risk scores and brain imaging measures. The age range was 45–81 years (mean 63.77).

### Genetic quality control

We used imputed genotype data released by the UK Biobank (March 2018). We excluded a random member of each pair of individuals with estimated kinship coefficient > 0.442 [65], participants with a mismatch of their self-reported and genetically inferred sex, putative sex chromosome aneuploidies, principle component-corrected heterozygosity > 0.19, or genotype missing rate > 0.05 [65]. Analyses were restricted to participants with “White British ancestry” as defined by Bycroft et al. [65]. We retained 9,803,522 bi-allelic variants with minor allele frequencies > 1%, INFO (imputation quality) score ≥ 0.7, and Hardy–Weinberg equilibrium *p*-value ≥ 10^−7^.

### Neuroimaging

Details of image processing and quality control are described elsewhere [66]. Measures of regional cortical surface area, cortical thickness, and subcortical volumes were derived from T1-weighted MRI scans (Siemens Skyra 3 Tesla MRI with 32-channel radio frequency receive head coil) and were released by the UK Biobank (February 2020, full protocol: http://biobank.ndph.ox.ac.uk/showcase/refer.cgi?id=2367). Briefly, Freesurfer 6.0 was used to parcellate the cerebral cortex into 34 regions per hemisphere according to the Desikan–Killiany atlas [67], and to segment 7 subcortical structures per hemisphere. Cortical surface area was measured at the gray–white matter boundary and cortical thickness was measured as the average distance in a region between the white matter and pial surfaces. Data for the temporal pole were excluded as unreliable [66]. In addition, separately per measure, we removed data points > 6 SDs from the mean.

We calculated the asymmetry index for each matching pair of the left and right measures, in each participant, as (left − right)/((left + right)/2), which is a commonly used formula for quantifying asymmetry [68, 69]. We then applied rank-based inverse normalization followed by linear regression to remove shared variance with potential confound variables, i.e., age, nonlinear age computed as (age-mean_age)^2^, the first ten principle components capturing genome-wide diversity in the genotype data [65], X-, Y-, and Z-scanner position parameters, T1 signal-to-noise ratio, T1 contrast-to-noise ratio, assessment center, genotyping array, and sex. We previously found that only 42 of the regional asymmetry indexes showed significant SNP-based heritabilities in this dataset (false discovery rate, FDR < 0.05) [51]. Accordingly, only these 42 asymmetry indexes were analyzed in the present study (Supplementary Table 1).

### Polygenic risks and brain asymmetry

We downloaded genome-wide, SNP-wise summary statistics from genome-wide association studies (GWASs) of ASD [29] (*n* = 46,350) and schizophrenia (*n* = 82,315). Separately for the two disorders, we used the PRS-CS software to calculate polygenic risk scores for each individual in the UK Biobank imaging dataset. PRS-CS uses a high-dimensional Bayesian regression framework to infer posterior effect sizes of SNPs, using genome-wide association summary statistics (i.e., for the 22 autosomes, excluding the sex chromosomes). We used default parameters and the recommended global effect size shrinkage parameter *φ* = 0.01, together with linkage disequilibrium information based on the 1000 Genomes Project phase 3 European-descent reference panel [70]. The ASD polygenic risk score was based on 1,092,064 autosomal SNPs and the schizophrenia score was based on 1,097,357 autosomal SNPs (these numbers came from the 3-way overlap between UK Biobank data, disorder genome scan data, and 1000 Genomes data). PRS-CS has been shown to perform highly similarly to other, recently developed polygenic risk methods for the prediction of psychiatric diseases and noticeably better than using linkage disequlibrium-based clumping with *P*-value thresholding [71]. PRS-CS has been validated using the same schizophrenia GWAS summary statistics as we used in the present study, applied to independent schizophrenia case–control data [71, 72].

Both polygenic risk scores were *z*-score scaled for subsequent analyses. Separately for the ASD and schizophrenia polygenic scores, canonical correlation analysis (“canoncorr” function in MATLAB: https://nl.mathworks.com/help/stats/canoncorr.html) was used to test their multivariate associations with the 42 brain asymmetry indexes across *N* = 32,256 individuals. This analysis found the linear combination of asymmetry indexes (i.e., the canonical asymmetry index) that was maximally associated with a given polygenic risk score across individuals.

For descriptive purposes, post hoc Pearson’s correlation analysis was performed between each polygenic score and each separate unilateral brain regional measure across *N* = 32,256 individuals (i.e., separately for the left and right measures, adjusted for the same covariate effects as above).

### Comparing multivariate patterns of brain asymmetry associated with polygenic risk for ASD and schizophrenia

The above canonical correlation analyses resulted in loadings, one value per brain regional asymmetry index, which reflected the extent and direction to which each asymmetry index drove the multivariate association with a given disorder polygenic risk score. The loadings were computed as correlations between each asymmetry index and the canonical variable representing all asymmetry indexes, across the 32,256 individuals.

We then calculated the correlation between the 42 loadings (1 per asymmetry index) derived from the analysis of the ASD polygenic risk score with the 42 loadings from the analysis of the schizophrenia polygenic risk score. This correlation (*N* = 42) indicated the extent to which polygenic risks for the two disorders were associated with similar or contrasting patterns of multivariate brain asymmetry. The empirical *p*-value of this correlation was calculated through 10,000 permutations in which within-subject relations between the two polygenic risk scores were maintained and within-subject relations between the 42 asymmetry indexes were maintained, but the polygenic risks were randomly shuffled with respect to the asymmetry indexes. This ensured that the *P*-value was robust to dependence between brain regions (various pairs of asymmetry indexes are moderately inter-correlated [51]).

### Functional annotation of brain regions

We identified asymmetry indexes that showed the strongest loadings with respect to ASD polygenic risk, as those indexes having loadings >0.2 or < −0.2. The bilateral brain regions corresponding to these asymmetry indexes were used to define a single mask encompassing all of these regions in standard space (Montreal Neurological Institute space 152). This mask was then analyzed using the “decoder” function of the Neurosynth database (http://neurosynth.org), a platform for large-scale synthesis of functional MRI data [73]. Neurosynth uses text-mining to detect frequently used terms as proxies for concepts of interest in the neuroimaging literature: terms that occur at a high frequency in a given functional imaging study are associated with all activation coordinates in that publication, allowing for automated term-based meta-analysis. We queried the database in December 2020 when it included 507,891 activation peaks reported in 14,371 studies. The input mask was first used to define a brain-wide co-activation map for that mask based on all studies in the database and the co-activation map was then correlated with each of the 1307 term-specific activation maps [73]. This analysis does not employ inferential statistical testing, but rather is designed to assess functional terms with respect to how strongly their meta-analyzed activation patterns correlate with a particular co-activation map derived from an input mask. We report only cognitive and disorder terms with correlation coefficients *r* > 0.2, while excluding anatomical terms, nonspecific terms (e.g., “Tasks”), and one from each pair of virtually duplicated terms (such as “Words” and “Word”). For these correlations, the sample size was 228,415 (the number of voxels brain-wide).

This analysis was also performed for the brain regional asymmetry indexes that showed the strongest loadings with respect to schizophrenia polygenic risk (i.e., again for those asymmetry indexes having loadings >0.2 or < −0.2).

### Analysis with respect to sex

We separately tested associations of ASD polygenic risk and schizophrenia polygenic risk with sex (as a binary integer) in the 32,256 individuals of the UK Biobank dataset, using two-sample *t*-tests.

We then used linear regression to adjust the 42 brain asymmetry indexes for potential confound effects across individuals as described above, except that sex was not included this time as a confound variable. For each of the 42 adjusted asymmetry indexes, we then performed a *t*-test with sex as the predictor variable (*N* = 32,256 individuals).

We then calculated the correlation between the 42 loadings (1 per asymmetry index) with respect to ASD polygenic risk and the 42 *t*-values (again 1 per asymmetry index) from the tests of association with sex. The sample size was therefore 42 for this correlation and its significance was assessed using permutations (*n* = 10,000) in which sex was randomly shuffled across individuals, to account for moderate non-independence between various pairs of asymmetry indexes. We further tested the correlation between the 42 loadings with respect to schizophrenia polygenic risk and the 42 *t*-values from the tests of association with sex (again significance was assessed using permutations (*n* = 10,000)). These analyses would reveal whether polygenic risk for either disorder was associated with an overall pattern of brain asymmetry resembling a more average male-like or female-like pattern.

### Handedness

Handedness was assessed by touchscreen questionnaire with four options: “Right-handed,” “Left-handed,” “Use both right and left hands equally,” and “Prefer not to answer.” We used the handedness data from the first visit of each individual to an assessment center. There were 28,703 right-handers, 3059 left-handers, and 490 mixed-handers among those with brain image and polygenic score data included in the present study. We used logistic regression with handedness groups as dependent variables (left-handed vs. right-handed, mixed-handed vs. right-handed, and non-right-handed vs. right-handed) to test associations with the ASD and schizophrenia polygenic risk scores, for a total of six separate logistic regressions. Covariates were the same as in the main analysis above.

### Phenome-wide association analysis of ASD and schizophrenia polygenic risks

The polygenic risk scores for ASD and schizophrenia were separately tested as predictor variables in phenome-wide association analysis (*N* = 32,256 individuals) using the PHESANT [74] software. The UK Biobank has collected diverse data on sociodemographic and lifestyle factors, psychological factors, imaging traits, cognitive functioning, and health. A selection of 3238 phenotypes of potential relevance to imaging genetics studies were available as part of our UK Biobank research application. Dependent variables of continuous, binary, ordered categorical, and unordered categorical types were tested using linear, logistic, ordered logistic, and multinominal logistic regression, respectively. Prior to testing, inverse normal rank transformation was applied to continuous variables. All analyses were adjusted for the same potential confounds as above, including sex. We excluded the specific brain phenotypes that were used to construct the regional asymmetry indexes in this study, as well as measures that were treated as covariates in the analysis, and we also excluded handedness, because this was already investigated separately (above). We controlled for multiple testing using an FDR threshold 0.05, separately for the two polygenic risk scores.

## Results

### Multivariate associations of brain asymmetry with polygenic risks for ASD and schizophrenia

Frequency histograms of the ASD and schizophrenia polygenic risk scores in the 32,256 participants from the UK Biobank are in Supplementary Fig. 1. There was a low but significant correlation between the two scores, *r* = 0.08, *p* = 7.13 × 10^−50^ (*N* = 32,256).

**Fig. 1:**
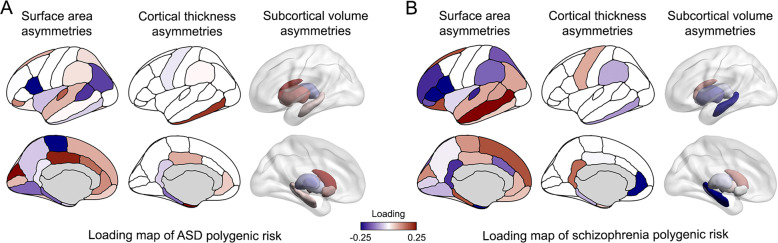
Map of associations of regional brain asymmetries with polygenic risks for ASD and schizophrenia. Loadings of regional brain asymmetries derived from canonical correlation analyses with ASD (**A**) and schizophrenia (**B**) polygenic risk scores. A positive loading (red) for a given region indicates a leftward shift of asymmetry associated with increased polygenic risk. Conversely, a negative loading (blue) indicates a rightward shift of asymmetry associated with increased polygenic risk.

Polygenic risk for ASD showed a significant multivariate association with the 42 regional brain asymmetry indexes, canonical correlation *r* = 0.03, *p* = 2.17 × 10^−9^ (*N* = 32,256). Polygenic risk for schizophrenia also showed a significant multivariate association with brain asymmetries, canonical correlation *r* = 0.04, *p* = 2.61 × 10^−11^ (*N* = 32,256). Loadings for each asymmetry index are in Supplementary Table 2 and Fig. 1A. A positive loading for a given regional asymmetry indicates a leftward shift associated with increased polygenic risk, for a given disorder. Conversely, a negative loading indicates a rightward shift of asymmetry associated with increased polygenic risk, for a given disorder.

There was no significant correlation (*r* = −0.03, permuted *p* = 0.87, *N* = 42) between the loadings across the 42 regional asymmetries for ASD polygenic risk and schizophrenia polygenic risk. This indicates that polygenic risks for ASD and schizophrenia have generally unrelated multivariate patterns of association with brain asymmetry. Only one asymmetry index, for the surface area of the pars opercularis (Fig. 1A), showed concordant loadings with respect to both of the polygenic risk scores (ASD loading −0.30 and schizophrenia loading −0.28), when concordance was assessed as having both loadings >0.2, or both < −0.2 (Supplementary Table 2). No asymmetry indexes with discordant loadings were observed, with loading >0.2 for one disorder polygenic risk and < −0.2 for the other. Results from post hoc univariate analysis of the separate left and right brain regional measures (adjusting for the same covariates as above) are in Supplementary Table 3.

### Functional annotation of regions driving multivariate associations with the two disorder PRS

**Fig. 2:**
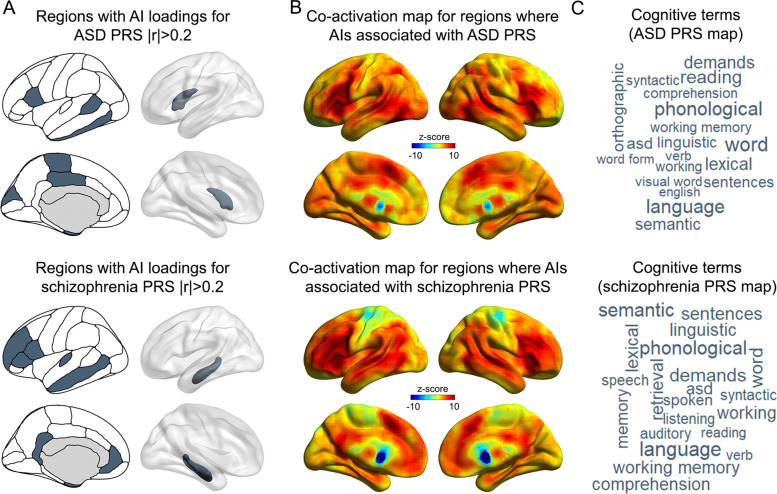
Functional annotation of regions showing the strongest associations with disorder polygenic risk scores (PRS). **A** Regions for which the AIs had loadings >0.2 in canonical correlation analysis with the ASD (top) and schizophrenia (bottom) polygenic risk scores, which were used to define binary masks to query the Neurosynth database. **B** Brain co-activation maps derived from the “decoder” function of Neurosynth, corresponding to the input masks for ASD (top) and schizophrenia (bottom) polygenic risks. **C** Word clouds of cognitive terms associated with the co-activation maps for ASD (top) and schizophrenia (bottom) polygenic risk scores. The font sizes of the terms indicate the correlations of their corresponding meta-analytic maps with the co-activation maps.

There were eight asymmetry indexes that showed loadings >0.2 or < −0.2 in multivariate analysis with ASD polygenic risk, distributed in temporal, inferior frontal, and posterior medial cortex, as well as the caudate nucleus volume asymmetry (Fig. 2A and Supplementary Table 2). The 16 regions (8 per hemisphere) corresponding to these 8 asymmetry indexes were used to create a single binary mask that was used to query the Neurosynth database of 14,371 functional brain imaging studies (see “Methods” and Fig. 2A). A brain-wide co-activation map (Fig. 2B) was generated for this mask, based on all functional maps in the database. There were 19 term-based correlations >0.2 with the co-activation map (*N* = 228,415 voxels brain-wide; Fig. 2C and Supplementary Table 4). The strongest of these was “word” (*r* = 0.37) and there were 14 other terms related to language in the list, such as “phonological” (*r* = 0.36), “language” (*r* = 0.34), and “reading” (*r* = 0.33). Three terms pertaining to executive functions were also in the list: “demands” (*r* = 0.30), “working memory” (*r* = 0.24), and “working” (*r* = 0.23), as well as the term “ASD” (*r* = 0.27; Supplementary Table 4).

We also created a mask corresponding to the nine regional asymmetry indexes that had shown loadings >0.2 or < −0.2 in multivariate analysis with schizophrenia polygenic risk. These were distributed over temporal, frontal, and cingulate cortex, plus the hippocampus (Fig. 2A). In the Neurosynth analysis, there were 21 correlations >0.2 (*N* = 228,415 voxels brain-wide; Fig. 2B, C and Supplementary Table 4) and these were again most prominently related to language (e.g., “language,” *r* = 0.33; “phonological,” *r* = 0.31) and executive functions (e.g., “demands,” *r* = 0.29; “working memory,” *r* = 0.27), and also again included the term “ASD” (*r* = 0.26) (Supplementary Table 4), despite that most regions were different in the binary masks for ASD polygenic risk and schizophrenia polygenic risk (Fig. 2).

### Brain asymmetry, disorder polygenic risk, and sex

Sex was associated with ASD polygenic risk (*N* = 32,256, *t* = 3.55, *p* = 3.84 × 10^−4^) and schizophrenia polygenic risk (*N* = 32,256, *t* = 2.77, *p* = 0.006), such that females tended to have higher polygenic risks for both disorders (despite that the risk scores were calculated from autosomal genotypes only) (Supplementary Fig. 2). This may reflect that participation in the UK Biobank was influenced by polygenic risks for these disorders in a sex-dependent manner (see “Discussion”).

**Fig. 3:**
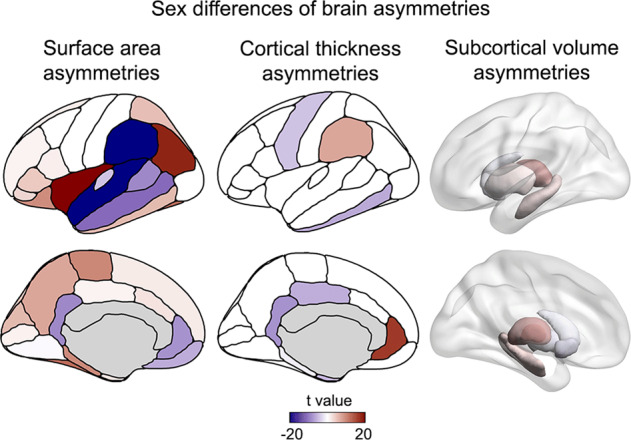
Sex differences of regional brain asymmetries. Sex differences of regional surface area asymmetries (left), cortical thickness asymmetries (middle), and subcortical volume asymmetries (right). A positive *t*-value (red) indicates an average leftward shift of asymmetry in females compared to males, whereas a negative *t*-value (blue) indicates an average leftward shift of asymmetry in males compared to females.

Sex was significantly associated with the majority of the brain regional asymmetry indexes across the 32,256 individuals (adjusted for all covariates as above, with the exception of sex) (Fig. 3, Supplementary Table 5, and Supplementary Fig. 3). In this analysis, a positive *t*-score for a given asymmetry index indicated an average rightward shift of asymmetry in males compared to females, and vice versa. The overall map of sex associations with regional brain asymmetries (Fig. 3) corresponded well with previous large-scale meta-analysis in other data [75].

The 42 *t*-values (1 per regional asymmetry index) from the sex-asymmetry association analysis were not significantly correlated with the 42 loadings from the ASD polygenic risk analysis (*N* = 42, *r* = −0.22, permuted *p* = 0.16) (where sex was controlled as one of the covariates in the latter). There was therefore no evidence that ASD polygenic risk was associated with an overall pattern of brain asymmetry more like either sex. Likewise, the 42 *t*-values (1 per regional asymmetry index) from the sex-asymmetry analysis were not significantly correlated with the 42 loadings from the schizophrenia polygenic risk analysis (*N* = 42, *r* = −0.13, permuted *p* = 0.50; Fig. 3) (where sex was again controlled as one of the covariates in the latter). This indicates that schizophrenia polygenic risk is also not associated with an overall pattern of brain asymmetry more like either sex.

### Handedness and polygenic risks for ASD and schizophrenia

There were 28,703 right-handers, 3059 left-handers, and 490 mixed-handers (therefore, 3549 non-right-handers). In logistic regression analysis, non-right-handedness was positively associated with ASD polygenic risk vs. right-handedness, *β* = 0.05, *p* = 0.004, driven particularly by an association of higher ASD polygenic risk with an increased rate of mixed-handedness (logistic regression *β* = 0.14, *p* = 0.002), rather than left-handedness (*β* = 0.04, *p* = 0.05), compared to right-handedness (Supplementary Fig. 4). Handedness was not associated with schizophrenia polygenic risk (all *p* > 0.05) (Supplementary Fig. 4).

### Phenome-wide association analyses of polygenic risk scores for ASD and schizophrenia

In phenome-wide association analysis of ASD polygenic risk, there were four significant associations at FDR 0.05 (Supplementary Table 6). The most significant was a positive association with “Hearing difficulty/problems with background noise” (*N* = 31,728, *β* = 0.06, adjusted *p* = 2.7 × 10^−3^), which was assessed through a touchscreen question “Do you find it difficult to follow a conversation if there is background noise (such as TV, radio, children playing).” The second most significant was a positive association with “Townsend deprivation index at recruitment” (*N* = 32,229, *β* = 0.03, adjusted *p* = 2.75 × 10^−3^). The third was a positive association with “Qualifications: College or University degree” (*N* = 32,054, *β* = 0.05, adjusted *p* = 5.55 × 10^−3^) and the fourth was a positive association with “Long-standing illness, disability or infirmity” (*N* = 31,751, *β* = 0.06, adjusted *p* = 1.53 × 10^−2^).

There were 495 significant associations with schizophrenia polygenic risk at FDR 0.05 (Supplementary Table 7). Most significant among these were associations of higher polygenic risk with poorer cognitive performance, e.g., “Interval between previous point and current one in alphanumeric path (trail #2)” (*N* = 20,948, *β* = 0.10, adjusted *p* = 2.13 × 10^−47^), and “fluid intelligence score” (*N* = 10,777, *β* = −0.14, adjusted *p* = 1.56 × 10^−14^). There were also significant associations in different directions with numerous brain regional gray and white matter metrics, as well as psychological factors (Supplementary Table 7). Although only ten individuals were diagnosed with schizophrenia (“Diagnoses—secondary ICD10: F20.9 Schizophrenia, unspecified”), this diagnosis was positively associated with schizophrenia polygenic risk (*N* = 32,255, *β* = 1.13, adjusted *p* = 0.0078) (Supplementary Fig. 5).

Full phenome-wide screening outputs (regardless of the FDR threshold 0.05) for the ASD and schizophrenia polygenic risk scores are in Supplementary Tables 6 and 7 (the sample sizes varied according to the availability of specific measures in the UK Biobank and are shown in these tables). “Volume of brain, gray + white matter” was not associated with ASD polygenic risk (*N* = 32,255, *β* = −0.006, uncorrected *p* = 0.16), but showed a negative association with schizophrenia polygenic risk (*N* = 32,255, *β* = −0.02, uncorrected *p* = 1.86 × 10^−5^, adjusted *p* = 3.58 × 10^−4^). As schizophrenia polygenic risk showed a negative association with brain size, we tested whether adjusting the 42 asymmetry indexes for brain size by linear regression across individuals (in addition to all other covariates used in the primary analysis above) would alter the multivariate associations of brain asymmetries with polygenic risk for either disorder. However, both multivariate associations remained significant and largely unchanged (ASD polygenic risk, canonical *r* = 0.03, *p* = 2.25 × 10^−9^, *N* = 32,256; schizophrenia polygenic risk, canonical *r* = 0.04, *p* = 2.57 × 10^−11^, *N* = 32,256).

## Discussion

In this study of the UK Biobank brain imaging-genetic dataset, we found that polygenic risks for ASD and schizophrenia were weakly correlated with each other, and each was significantly associated with a distinct multivariate loading pattern across brain asymmetry measures. There was no evidence that multivariate patterns of brain asymmetry associated with these two polygenic risks were anti-correlated and, therefore, our results do not support concepts of ASD and schizophrenia as opposing disorders on a single neurobiological dimension (see “Introduction”). Rather, our findings support ASD and schizophrenia as largely, but not wholly, distinct disorders at the genetic and neurobiological levels.

Neither polygenic risk score was associated with a more male-like or female-like pattern of brain asymmetry, after adjusting the asymmetry indexes for sex. Thus, at least with respect to structural brain asymmetry, ASD polygenic risk does not appear to resemble increased masculinization, as might be expected according to the “extreme male brain” theory of ASD [2]. Previous studies that directly assessed brain masculinization in ASD have focused primarily on functional connectivity using resting-state functional brain imaging data and have reported both increased and decreased masculinization in ASD individuals that may be network-specific [76–78]. In addition, no convincing relationship of prenatal androgen exposure has been found in relation to autistic traits [79, 80]. Comparisons of cognitive profiles of ASD and schizophrenia to average sex differences may therefore be superficial, rather than reflecting an underlying risk dimension of etiological relevance.

The single asymmetry index that was noticeably concordant in its associations with both polygenic risks was that of the pars opercularis surface area, a region of the inferior frontal cortex that forms part of Broca’s classically defined language-production region [81]. Language is a left-lateralized function in most people and some leftward structural asymmetries of language-important regions, such as the pars opercularis, may reflect this functional laterality at the population level [82]. The pars opercularis showed reduced leftward asymmetry with higher polygenic risk for both disorders, consistent with leftward asymmetry being the optimal structural organization. This region is likely to have contributed to the language-related terms that we observed for both polygenic risk scores, in functional annotation of regions for which asymmetries were associated with the risk scores. Therefore, the two disorders may share an etiological link through altered structure and function of Broca’s region, consistent with impaired social communication in both disorders (see “Introduction”). Altered hemispheric asymmetry of regions important for language has been proposed to contribute to auditory verbal hallucinations in schizophrenia [36, 83], perhaps due to disrupted fronto-temporal connectivity in the left hemisphere, leading to a failure of top-down control of bottom-up perceptual processes [84].

In addition to language, functional annotation indicated that brain regions for which asymmetries were associated with polygenic risks for the two disorders were involved in executive functions. This was consistent with the phenome-wide association results, where a higher polygenic risk for schizophrenia was most prominently associated with lower performance on tests requiring executive function (e.g., symbol-digit substitution and trail making). In contrast, ASD polygenic risk was significantly and positively associated with college or university qualifications, and also showed a positive trend of association with fluid intelligence (*β* = 0.04, uncorrected *p* = 8.78 × 10^−4^), although the latter did not meet FDR correction at 0.05. A positive genetic correlation between ASD and educational attainment has been reported previously [18]. These observations support the validity of the polygenic risk scoring in the UK Biobank data. A recent meta-analysis that compared cognitive performance across multiple domains in ASD and schizophrenia, in adulthood, also found the clearest differences in visuospatial perception, reasoning, and problem solving, with ASD individuals performing better [85]. ASD polygenic risk was most significantly associated with difficulties following conversation in the presence of background noise, which may relate to a linguistic or social cognitive difficulty, rather than a sensory issue. Taken all together, the patterns of polygenic risk–brain behavior associations in the UK Biobank suggest that largely distinct alterations of regional brain asymmetry may be functionally and etiologically relevant manifestations of high polygenic risks for ASD and schizophrenia, although future analyses in longitudinal data will likely be required to help clarify mediation and cause–effect relations.

The association effect sizes in this study were small, with canonical correlations no greater than 0.04, and the high degree of statistical significance arose through the large sample size. Macro-structural measures of regional asymmetry are relatively crude biological readouts, but it is possible that more substantial associations of ASD and schizophrenia polygenic risks will be detected with brain asymmetries measured at higher levels of resolution, e.g., in the abundances of specific cell or synaptic types, or gene expression levels, with potentially more direct functional relevance. The brain asymmetry measures in this study all had heritabilities <10%, which means that any polygenic effects will necessarily be limited. In fact, most variance in brain asymmetries may be due to early developmental randomness [51, 86–88]. The value of the present findings is not in terms of biomarkers for disorder risk, but rather in terms of biological clues into potentially relevant aspects of disorder etiology.

We found no evidence that brain size influenced the associations of either polygenic risk with brain asymmetries. This may be expected, as the asymmetry index (left − right)/((left + right)/2) is adjusted through its denominator for the bilateral magnitude of any given regional measure. We did observe that schizophrenia polygenic risk was associated with reduced brain size, consistent with average reductions in cortical surface area and thickness, and subcortical volumes, which have been reported to associate with schizophrenia by large-scale consortium analysis [89, 90]. There was no association of ASD polygenic risk with brain size. This may reflect that the average age of the UK Biobank individuals in this study was 63 years. A previous study of 259 ASD individuals and 327 controls, aged up to 50 years, found relative brain overgrowth in ASD during infancy and childhood, followed by accelerated decline in size from adolescence onwards [91]. It is a limitation of the present study that the age of UK Biobank individuals is far older than the typical ages of onset of either ASD or schizophrenia. However, both disorders often persist throughout life and it is during adulthood that they can be most directly compared and contrasted in terms of affected brain traits and cognitive profiles.

Polygenic risks for ASD and schizophrenia were weakly and positively correlated with each other in the present study (*r* = 0.08), which is broadly consistent with a positive genetic correlation of roughly 0.2 between the two disorders [18], and supports the validity of the polygenic risk scores in the UK Biobank data. Both polygenic risks were also slightly higher in females than in males. The UK Biobank is not fully representative of the population, with generally superior health and socioeconomic circumstances, and greater female participation [92]. Higher risk for psychiatric disorders has also been suggested to reduce participation in cohort studies [93]. Together, these observations suggest that the UK Biobank particularly under-represents males with high polygenic risks for ASD and schizophrenia. Regardless, when adjusting for sex, the multivariate associations of both polygenic risks with brain asymmetries were highly significant and not associated with either a more male-like or more female-like average pattern.

Schizophrenia polygenic risk was positively associated with the UK Biobank trait “Diagnoses—secondary ICD10: F20.9 Schizophrenia, unspecified.” However, only ten participants had this diagnosis and there is not a similar phenotype recorded for autism. This meant that the polygenic risk scores could not be validated against diagnoses in this dataset. In general, larger sample sizes for case–control genome-wide association scans yield more accurate summary statistics for input into polygenic scoring methods, thus producing more accurate polygenic risk scores. The ASD polygenic score may therefore have been less accurate than the schizophrenia polygenic score (see “Methods”). Any inaccuracy in the polygenic scores may have reduced their inter-correlation and their associations with brain and other phenotypes in this study. Furthermore, polygenic scores only index the risk for having a disorder that is attributable to common genetic variation, rather than being direct indexes of case–control status. For example, the odds ratio for a schizophrenia diagnosis in electronic health record data, per SD increase in schizophrenia polygenic risk, was estimated at 1.55 (95% confidence interval = 1.4, 1.7) in a study that used the same polygenic risk calculation method (PRS-CS) and case–control genome-wide scan summary statistics as the present study [72]. Performance with respect to diagnosis, brain traits, and other associated phenotypes may be improved, as larger case–control genome-wide association scans are published.

We did not consider handedness as a confound variable in our analyses, because we were interested in associations of disorder polygenic risks with structural brain asymmetries regardless of their associations with other aspects of brain or behavioral asymmetry. Handedness may itself be a causally influenced variable by both disorder polygenic risks and brain structural asymmetry; thus, treating handedness as a confound variable could have induced collider bias into our analysis [94]. There was evidence that ASD polygenic risk was positively associated with non-right-handedness, and in particular mixed-handedness, consistent with an increased rate of these traits in ASD [41]. This observation further supports the validity of the ASD polygenic risk scoring in the UK Biobank data. We did not detect an association of schizophrenia polygenic risk with handedness.

In summary, we found that polygenic risks for ASD and schizophrenia have generally distinct associations with brain asymmetries. Findings were consistent with a particular involvement of regions important for language and executive functions, but there was no evidence that the two polygenic risks were associated with overall opposing patterns of brain asymmetry or with excessive masculinization or feminization of the brain. The most concordant association for the two polygenic risks involved asymmetry of the pars opercularis, which supports the two disorders as sharing etiological features in the language domain. These findings contribute to an improved understanding of potentially shared and distinct etiological risks for ASD and schizophrenia.

## Supplementary information

Supplemental material

Supplementary tables

## Data Availability

The primary data used in this study are available via the UK Biobank website www.ukbiobank.ac.uk. Other publicly available data sources and applications are cited in the manuscript.

## Code availability

This study used openly available software and codes as cited in the “Methods” section.
