# Supplementary material for: Patterns of brain asymmetry associated with polygenic risks for autism and schizophrenia implicate language and executive functions but not brain masculinization
Source: Mol Psychiatry. 2021 Jul 1;26(12):7652–60. doi: 10.1038/s41380-021-01204-z (PMC8872997; doi:10.1038/s41380-021-01204-z)
Supplement: Supplementary file 1 — Supplemental material [file 41380_2021_1204_MOESM1_ESM.pdf]

Supplementary information

Sha et al. Patterns of brain asymmetry associated with polygenic risks for autism and schizophrenia implicate language and executive functions but not brain masculinization

Contents

Supplementary Figures 1-5, pages 2-6.

Supplementary Tables 1-7: See separate spreadsheet file for supplementary tables.

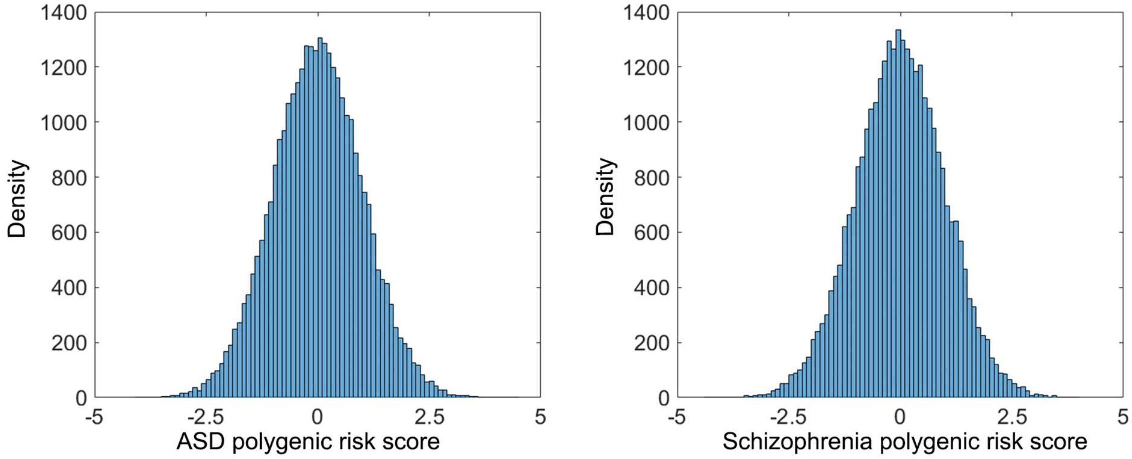

Supplementary Figure 1. Frequency histograms of the ASD and schizophrenia polygenic risk scores in 32,256 participants of the UK Biobank brain imaging genetics dataset.

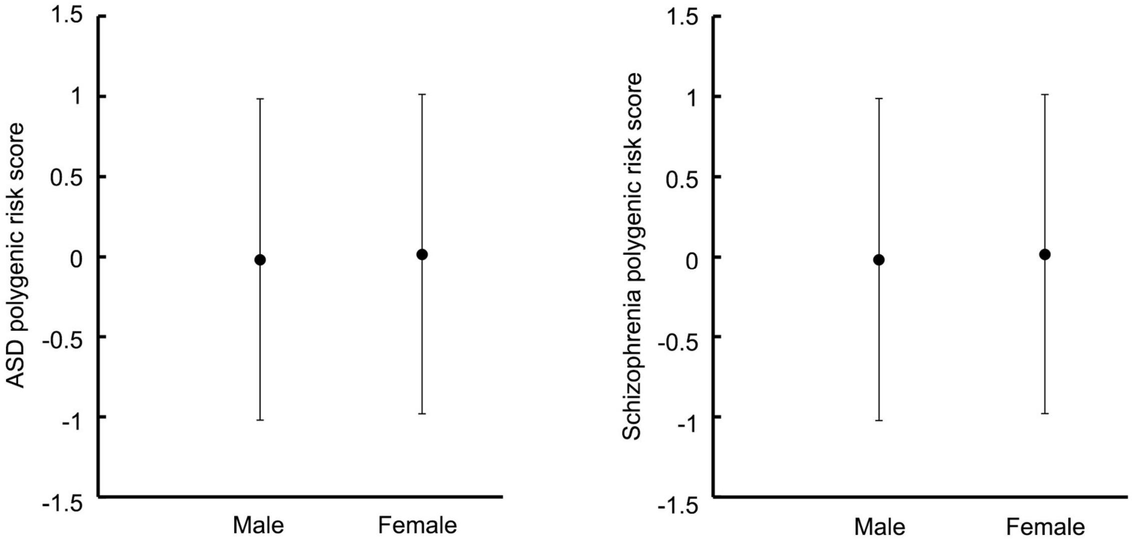

Supplementary Figure 2. Means and standard deviations of ASD and schizophrenia polygenic risk scores, separately in males and females.

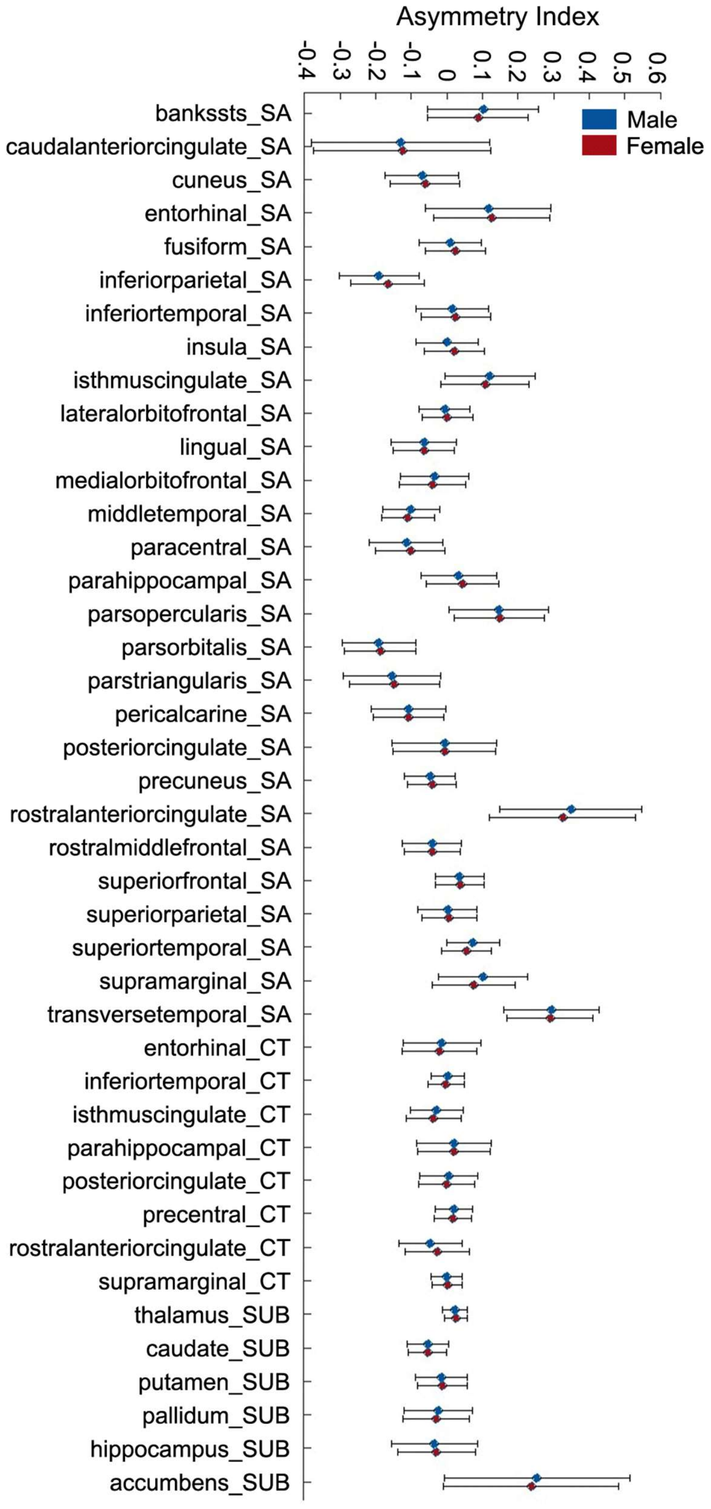

Supplementary Figure 3. Means and standard deviations of brain regional asymmetry indexes, separately in males and females. Abbreviations: SA: surface area; CT: cortical thickness; SUB: subcortical volume.

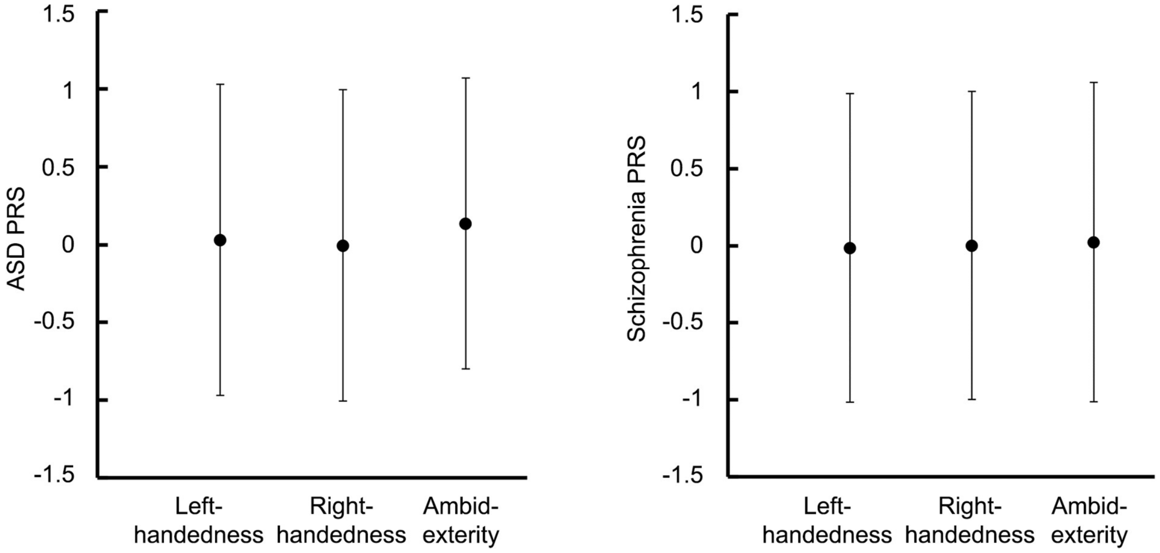

Supplementary Figure 4. Means and standard deviations of polygenic risk scores for ASD and schizophrenia, plotted separately by handedness groups.

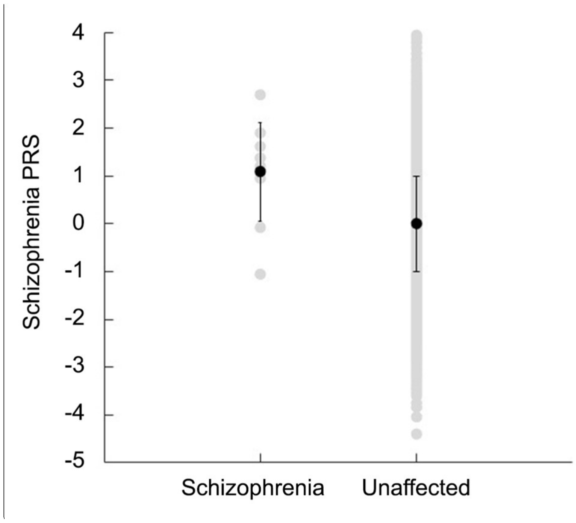

Supplementary Figure 5. Polygenic risk scores (PRS) for schizophrenia in 10 individuals with the diagnosis 'secondary ICD10: F20.9 Schizophrenia, unspecified', versus 32,245 unaffected individuals in the dataset. Individuals are shown by grey circles, means and standard deviations in black.
